# Development of a fluorogenic ADAMTS-7 substrate

**DOI:** 10.1080/14756366.2021.1983808

**Published:** 2021-09-30

**Authors:** Salvatore Santamaria, Frederic Buemi, Elisa Nuti, Doretta Cuffaro, Elena De Vita, Tiziano Tuccinardi, Armando Rossello, Steven Howell, Shahid Mehmood, Ambrosius P. Snijders, Rens de Groot

**Affiliations:** a Department of Immunology and Inflammation, Imperial College London, London, UK; b Department of Pharmacy, University of Pisa, Pisa, Italy; c Proteomics Science Technology Platform, The Francis Crick Institute, London, UK; d Institute of Cardiovascular Science, University College London, London, UK

**Keywords:** ADAMTS-7, ADAMTS7, activity assay, inhibitor, coronary artery disease

## Abstract

The extracellular protease ADAMTS-7 has been identified as a potential therapeutic target in atherosclerosis and associated diseases such as coronary artery disease (CAD). However, ADAMTS-7 inhibitors have not been reported so far. Screening of inhibitors has been hindered by the lack of a suitable peptide substrate and, consequently, a convenient activity assay. Here we describe the first fluorescence resonance energy transfer (FRET) substrate for ADAMTS-7, ATS7FP7. ATS7FP7 was used to measure inhibition constants for the endogenous ADAMTS-7 inhibitor, TIMP-4, as well as two hydroxamate-based zinc chelating inhibitors. These inhibition constants match well with IC_50_ values obtained with our SDS-PAGE assay that uses the N-terminal fragment of latent TGF-β–binding protein 4 (LTBP4S-A) as a substrate. Our novel fluorogenic substrate ATS7FP7 is suitable for high throughput screening of ADAMTS-7 inhibitors, thus accelerating translational studies aiming at inhibition of ADAMTS-7 as a novel treatment for cardiovascular diseases such as atherosclerosis and CAD.

## Introduction

The extracellular protease A Disintegrin-like And Metalloproteinase with Thrombospondin Motif (ADAMTS)-7 has been strongly implicated in the aetiology of atherosclerosis and coronary artery disease (CAD). A genomewide association study (GWAS) has demonstrated an inverse association between atherosclerosis and a non-synonymous single-nucleotide polymorphism (SNP), leading to a Ser-to Pro substitution in the prodomain of ADAMTS-7^1^. This aminoacid substitution interferes with ADAMTS-7 secretion and activation by proprotein convertases such as furin, suggesting that decreased ADAMTS-7 activity may be beneficial for the treatment of atherosclerosis^1^^,^^2^. Other GWASs have identified SNPs in the genomic region of ADAMTS-7 as risk loci for CAD^3^^,^^4^. Human atherosclerotic plaques are also enriched in ADAMTS-7^1^^,^^5^. The detrimental role exerted by ADAMTS-7 in cardiovascular diseases has been corroborated by *in vivo* models. Mice bearing a null or inactivating mutation for *Adamts7* showed a significant reduction in the size of atherosclerotic lesions compared to littermate controls^2^^,^^6^. *Adamts7* null mice also showed reduced neointima formation upon vascular injury^2^^,^^6^^,^^7^. Importantly, ADAMTS-7 catalytic activity is required for optimal vascular smooth muscle cell migration^2^. These findings suggest that pharmacological inhibition of ADAMTS-7 activity could slow down the progression of atherosclerosis and associated CAD. However, no inhibitors of ADAMTS-7 have been described so far. A major obstacle for inhibitor development is the lack of a suitable activity assay which can be adapted for high-throughput screening (HTS) of large molecule libraries, the most common drug discovery approach in the absence of a lead compound. Fluorescence resonance energy transfer (FRET) substrates are best suited for this purpose. However, FRET substrates designed for ADAMTS-4 and ADAMTS-5 were not efficiently cleaved by ADAMTS-7 so the design of a new FRET substrate was required.

Recently, using terminal amine isotopic labelling of substrates, we have identified a novel substrate for ADAMTS-7, latent TGF-β–binding protein (LTBP)4^8^. Proteolysis of LTBP4 by ADAMTS-7 occurs in a linker region between the first EGF-like domain and the hybrid domain^8^. The identification of the scissile bonds has allowed us to design a short peptide substrate suitable for assays based on FRET. Here, we describe the first FRET substrate for ADAMTS-7, based on ADAMTS-7 cleavage sites in LTBP4. This new FRET substrate is cleaved efficiently by ADAMTS-7, ADAMTS-4 and ADAMTS-5, but not appreciably by ADAMTS-1 or ADAMTS-8. Importantly, it provides a rapid platform to identify ADAMTS-7 inhibitors. As a proof of concept, we used the new substrate to measure inhibition constants for tissue inhibitor of metalloproteinase (TIMP)-4, an endogenous ADAMTS-7 inhibitor^8^, as well as two novel hydroxamate-based zinc-chelating inhibitors. We showed that inhibition constants measured with the FRET substrate correlated well with IC_50_ values measured using LTBP4. Importantly, our novel assay can be used for HTS of large molecule libraries to identify ADAMTS-7 inhibitors.

## Materials and methods

### Recombinant proteins and FRET peptides

All FRET peptides reported in this study were custom-synthesised by Bachem (Switzerland). Aliphatic index and Grand average of hydropathicity (GRAVY) were computed using ProtParam (https://web.expasy.org/protparam/). Recombinant human pro-MMP-2 and ADAM-17 were purchased from Calbiochem (Merck-Millipore). Pro-MMP-12 was purchased from R&D Systems (Cat. n.: 917-MP). Expression and purification of recombinant human ADAMTS-1, −4, −5 and −7 have been reported before^8–10^. ADAMTS-7 used in this study was the recombinant construct ADAMTS7-T8, which lacks the C-terminal PLAC domain and is described in Colige et al^8^ and de Groot et al^10^. ADAMTS-8 was transiently expressed in HEK293T and purified with anti-FLAG M2 affinity agarose (Sigma Cat. n: A2220) exactly like ADAMTS-1, ADAMTS − 4 and ADAMTS − 5. ADAMTS-1, ADAMTS − 4, ADAMTS − 5 and ADAMTS − 8 constructs had a C-terminal FLAG-tag (Asp-Tyr-Lys-Asp-Asp-Asp-Asp-Lys). Enzyme concentrations were determined by active site titration^9^^,^^11^. The mammalian expression vector for LTBP4S-A, containing an N-terminal FLAG tag was a generous gift of Tomoyuki Nakamura (Kansai Medical University, Osaka, Japan) and details of its generation have been described previously^12^. Recombinant human TIMP-4 was purchased from R&D (Cat. n: 974-TSF).

#### Synthesis of EDV33

##### General

All reactions were performed in solution, mostly under N_2_ atmosphere (or Ar where specified) in dried glassware and were followed by thin layer chromatography (TLC) on Merck aluminium silica gel sheets (60 F_254_) visualised under a UV lamp (254 nm; 365 nm). Evaporation was conducted under vacuum condition in rotating evaporator. Anhydrous sodium sulphate was used as a drying agent after extraction treatments. Purification was achieved either by trituration with *n*-hexane and Et_2_O or liquid chromatography. Chromatographic separations were performed on silica gel by flash columns (Kieselgel 40, 0.040–0.063 mm; Merck) or by ISOLUTE Flash Si II cartridges (Biotage). ^1^H, ^13 ^C and ^19 ^F NMR spectra were recorded on a Bruker Avance III HD 400 MHz spectrometer. Chemical shift (δ) are reported in ppm and *J* in Hz. The following abbreviations were used to explain the multiplicities: s = singlet, d = doublet, t = triplet, q = quartette, m = multiplet, dt = double triplet, dd = double doublet, ds = double septet, br s = broad singlet. Melting points were determined on a Kofler hotstage apparatus and are uncorrected. All commercially available chemicals were purchased from Sigma-Aldrich. Elemental analysis has been used to determine the purity of target compound. Analytical results are within ±0.40% of the theoretical values.

###### (R)-5-((tert-Butoxycarbonyl)amino)-2–(4-((2-chloro-4-fluorobenzyl)oxy)phenylsulphonamido) pentanoic acid (2)

To a solution of (*R*)-*N*-Boc-ornithine (1.34 mmol, 0.31 g) in H_2_O/dry dioxane 1:1 v/v (2.72 ml) under nitrogen atmosphere, triethylamine (2.69 mmol, 0.38 ml) and 4-((2-chloro-4-fluorobenzyl)oxy)benzene-1-sulphonyl chloride (1) (1.34 mmol, 0.50 g) were added. The mixture was stirred overnight, then diluted with EtOAc (100 ml) and washed with 1 N HCl (80 ml). The aqueous phase was also extracted with EtOAc (200 ml). The reunited organic phases were washed with brine (150 ml), dried over Na_2_SO_4_ and evaporated *in vacuum* to give compound **2** (0.70 g) as a white solid. Yield: 99.6%. ^1^H NMR (MeOD) δ: 1.44 (*s*, 9H); 1.45–1.80 (*m*, 4H); 3.01 (dt, *J_1_* = 6.7 Hz, *J_2_* = 1.8 Hz, 2H); 3.80–3.84 (*m*, 1H); 5.23 (*s*, 2H); 7.12–7.18 (*m*, 3H); 7.32 (dd, *J_o_* = 8.6 Hz, *J_m_* = 2.6 Hz, 1H); 7.60–7.64 (*m*, 1H); 7.78–7.89 (*m*, 2H). ^13 ^C NMR (100 MHz, MeOD) δ: 25.6; 27.4; 29.9; 39.2; 55.5; 68.1; 78.5; 115.3 (d, *J*_2 C-F_ = 21.2 Hz); 116.1; 117.9 (d, *J*_2 C-F_ = 25.12 Hz); 130.4; 131.6 (d, *J*_4 C-F_ = 3.5 Hz); 132.5 (d, *J*_3 C-F_ = 8.7 Hz); 134.7; 135.5 (d, *J*_3 C-F_ = 10.6 Hz); 157.1; 163.2; 163.9 (d, *J*_1 C-F_ = 247.5 Hz); 173.6.

###### tert-Butyl ((4 R)-4–(4-((2-chloro-4-fluorobenzyl)oxy)phenylsulphonamido)-5-oxo-5-((((tetrahydro-2H-pyran-2-yl)oxy)amino)oxy)pentyl)carbamate (3)

1-Hydroxybenzotriazole hydrate (1.58 mmol, 0.21 g), *N*-methylmorpholine (3.96 mmol, 0.44 ml), (tetrahydro-2H-pyran-2-yl)hydroxylamine (4.09 mmol, 0.47 g) and lastly *N*-(3-dimethylaminopropyl)-*N*'-ethylcarbodiimide hydrochloride (1.84 mmol, 0.35 g) were added to a solution of compound **2** (1.32 mmol, 0.70 g) in dry DMF (3.45 ml). The mixture was stirred overnight under argon, then diluted with EtOAc (150 ml). This solution was washed with H_2_O (80 ml), a saturated solution of NaHCO_3_ (60 ml) and brine (60 ml) then dried over Na_2_SO_4_ and evaporated. The residue was purified by ISOLUTE chromatography (Si II, 10 g; CH_2_Cl_2_) to give compound **3** (0.40 g) as a white-pink solid. Yield: 49.2%. ^1^H NMR *diastereoisomeric mixture* (CDCl_3_) δ: 1.43 (*s*, 9H); 1.44 (*s*, 9H); 1.46–1.90 (*m*, 20H); 2.99–3.04 (*m*, 2H); 3.37–3.67 (*m*, 4H); 3.80–4.05 (*m*, 4H); 4.36 (br s. 1H); 4.78 (br s, 1H); 5.16 (*s*, 4H); 5.46 (d, *J* = 9.6 Hz, 1H); 5.61 (d, *J* = 9.2 Hz, 1H); 7.02–7.07 (*m*, 6H); 7.17–7.21 (*m*, 2H); 7.49–7.54 (*m*, 2H); 7.79–7.82 (*m*, 4H); 9.38 (br s, 1H); 9.62 (br s, 1H).

###### (4R)-4–(4-((2-chloro-4-fluorobenzyl)oxy)phenylsulphonamido)-5-oxo-5-((((tetrahydro-2H-pyran-2-yl)oxy)amino)oxy)pentan-1-aminium trifluoroacetate (4)

Trifluoroacetic acid (3.17 mmol, 0.24 ml) was added dropwise to a solution of compound **3** (0.15 mmol, 0.10 g) in dry CH_2_Cl_2_ (1 ml) cooled at 0 °C. The mixture was stirred at 0 °C under nitrogen atmosphere for 30 min. The acid excess was removed by trituration with *n*-hexane and the crude product was purified by ISOLUTE chromatography (Si II, 5 g; CHCl_3_/MeOH 15:1 v/v). Compound **4** (0.04 g) was obtained as a colourless oil. Yield: 40.7%. ^1^H NMR *diastereoisomeric mixture* (DMSO-*d*_6_) δ: 1.33–1.83 (*m*, 20H); 2.68–2.75 (*m*, 4H); 3.60–3.68 (*m*, 2H); 3.74–3.92 (*m*, 4H); 4.23–4.27 (*m*, 1H); 4.66–4.69 (*m*, 1H); 5.13–5.23 (*m*, 4H); 7.10–7.20 (*m*, 4H); 7.26–7.83 (*m*, 2H); 7.52–7.58 (*m*, 2H); 7.65–7.75 (*m*, 6H); 7.76–7.81 (*m*, 1H); 7.96–8.06 (*m*, 1H); 11.09 (*s*, 1H); 11.21 (*s*, 1H).

###### N-((4R)-4–(4-((2-chloro-4-fluorobenzyl)oxy)phenylsulphonamido)-5-oxo-5-((((tetrahydro-2H-pyran-2-yl)oxy)amino)oxy)pentyl)benzamide (5)

Compound **4** (0.14 mmol, 0.09 g) was dissolved in dry DMF (1.5 ml) under nitrogen atmosphere, then *N*,*N*-diisopropylethylamine (0.28 mmol, 0.05 ml) and benzoyl chloride (0.17 mmol, 0.02 ml) were added and the mixture was stirred overnight. The solution so obtained was diluted with H_2_O (30 ml) and extracted with EtOAc (150 ml), which was then washed with brine (80 ml) and dried over Na_2_SO_4_. After evaporation *in vacuum*, the residue was purified by ISOLUTE chromatography (Si II, 5 g; *n*-hexane/EtOAc 3:1 v/v) to give compound **5** (0.05 g) as a yellow solid. Yield: 64.8%. ^1^H NMR *diastereoisomeric mixture* (CDCl_3_) δ: 1.55–2.08 (*m*, 20H); 3.28–3.40 (*m*, 4H); 3.44–3.53 (*m*, 2H); 3.85–4.00 (*m*, 4H); 4.39 (br s, 1H); 4.77–4.82 (*m*, 1H); 5.17 (*s*, 4H); 5.61–5.66 (*m*, 1H); 5.75–5.80 (*m*, 1H); 6.58–6.66 (*m*, 2H); 7.00–7.10 (*m*, 4H); 7.18–7.24 (*m*, 2H); 7.42–7.59 (*m*, 6H);7.74–7.90 (*m*, 12H); 9.84 (br s, 1H); 9.90 (br s, 1H).

###### (R)-N-(4–(4-((2-chloro-4-fluorobenzyl)oxy)phenylsulphonamido)-5-((hydroxyamino)oxy)-5-oxopentyl)benzamide (EDV33)

4 N HCl in dioxane (1.71 ml) was added dropwise to a solution of compound **5** (0.09 mmol, 0.05 g) in dioxane (1.5 ml). MeOH was then added (0.5 ml) to dissolve the suspension and the mixture was stirred under nitrogen for 1.5 h. After evaporation *in vacuum*, the product was triturated with Et_2_O to give final compound EDV33 (0.03 g) as a white solid. Yield: 71.5%, Mp: 187–189 °C (Et_2_O) (Supplementary Figures 1–3). ^1^H NMR (400 MHz, DMSO-*d*_6_) δ: 1.38–1.53 (*m*, 4H); 3.10–3.17 (*m*, 2H); 3.52–3.58 (*m*, 1H); 5.18 (*s*, 2H); 7.13–7.20 (*m*, 2H); 7.29 (dt, *J_o_* = 8.5 Hz, *J_m_* = 2.8 Hz, 1H); 7.42–7.52 (*m*, 3H); 7.54 (dd, *J_o_* = 8.8 Hz, *J_m_* = 2.6 Hz, 1H); 7.66–7.70 (*m*, 1H); 7.71–7.76 (*m*, 2H); 7.82–7.85 (*m*, 2H); 7.89 (d, *J* = 8.5 Hz, 1H); 8.42 (*t*, *J* = 2.2 Hz, 1H); 8.83 (br s, 1H); 10.55 (*s*, 1H). ^19 ^F NMR (376 MHz, DMSO-*d*_6_) δ: −111.30. ^13 ^C NMR (100 MHz, DMSO-*d*_6_) δ: 25.9; 30.9; 39.1; 54.2; 67.1; 115.0 (d, *J_2_*_C-F_ = 21.2 Hz); 115.3; 117.3 (d, *J_2_*_C-F_ = 25.1 Hz); 127.5; 128.6; 129.0; 130.6 (d, *J_4_*_C-F_ = 3.5 Hz); 131.4; 132.5 (d, *J_3_*_C-F_ = 9.3 Hz); 134.2; 134.3 (d, *J_3_*_C-F_ = 10.5 Hz); 135.0; 161.2; 162.3 (d, *J_1_*_C-F_ = 248.1 Hz); 166.5; 167.7. Elemental Analysis for C_25_H_25_ClFN_3_O_6_S, calculated: % C, 54.59; % H, 4.58; % N, 7.64. Found: % C, 54.72; % H, 4.63; % N, 7.51 .

### Determination of inhibition constants

All enzyme assays were conducted in TNC-B buffer (50 mM Tris- HCl, pH 7.5, 150 mM NaCl, 10 mM CaCl_2_, and 0.02% NaN_3_) at 37 °C in 384 well plates (Cat. No: 784900, Greiner Bio-One, Austria). To avoid the formation of inhibitor or peptide aggregates, the detergent Brij-35 (0.05%) was added to TNC-B^13^. For inhibition studies, stock solutions of chemical compounds were prepared in dimethyl sulfoxide (DMSO) (10 mM). These were further diluted at different concentrations in TNC-B and incubated with ADAMTS-1, ADAMTS-4, ADAMTS-5, ADAMTS-7 or ADAMTS-8 (10 nM) for 1 h at 37 °C. Blank samples were prepared using buffer only and the rate of cleavage for blank reactions was subtracted from the rates measured in the presence of enzyme. Upon addition of the indicated ADAMTS-7 FRET substrates (40 µM), the fluorescence was measured immediately using a FLUOstar Omega microplate reader (BMG Labtech, Germany) with an excitation filter wavelength of 485 nm and an emission filter wavelength of 520 nm.

Concentrations of other metzincins were 0.5 nM (MMP-2), 2.3 nM (MMP-12) and 5 nM (ADAM-17) and the following FRET substrate was used: Mca-Lys-Pro-Leu-Gly-Leu-Dap(Dnp)-Ala-Arg-NH_2_ (Bachem) (2 µM), with an excitation filter wavelength of 325 nm and an emission filter wavelength of 400 nm. Activation of proenzymes was carried out as previously described^14^^,^^15^.

Percent of inhibition was calculated from control reactions containing only DMSO. IC_50_ values were determined using the formula:

$$v_{i}/v_{0}=1/\left(1\;+\;\left[I\right]/{\text{IC}}_{50}\right)$$

where *v*_i_ is the initial velocity of substrate cleavage in the presence of the inhibitor at concentration [I] and *v*_0_ is the initial velocity in the presence of an equal concentration (v/v) of DMSO. For zinc-chelating small molecule inhibitors, *K*_i app_ values were determined using the Cheng-Prusoff equation^16^:

$$K_{i\;\text{app}}={\;\text{IC}}_{50}/\left(1+\left[S\right]/K_{m}\right)$$

where [S] = 40 µM and *K*_m_ = 10.5 µM for ADAMTS-7. For the other metzincins, substrate and *K*_m_ values used to determine *K*_i app_ values are reported in Supplementary Table 1. In case of TIMP-4, the *K*_i_ value was of the same order of magnitude as the enzyme concentration in the reaction mixture, so *K*_i_ values were determined using the Morrison equation for tight binding inhibitors^17^:

$$v_{i}/v_{0}=\;1-(({\left[E\right]}_{t}+\left[I\right]+K_{i\;\text{app}})-\surd ({\left({\left[E\right]}_{t}+\left[I\right]+K_{i\;\text{app}}\right)}^{2}-4{\left[E\right]}_{t}\left[I\right])/2\left[E{]}_{t}\right)$$

where [E]_t_ is the total active enzyme concentration.

### Determination of kinetic constants for cleavage of ATS7FP7

*K*_m_ values were determined at increasing concentrations (0–160 µM) of ATS7FP7 in the presence of 10 nM ADAMTS-7 by nonlinear fitting to the Michaelis-Menten equation:

$$v_{0}={\;V}_{\text{max}}\left[S\right]/\left(K_{m}+\;\left[S\right]\right)$$

where V_max_ is the maximum initial velocity for the reaction. Relative Fluorescence Units (RFU) were converted into product concentration by dividing for the number of RFU generated upon complete digestion of 2.5 µM substrate. *k*_cat_ values were determined by dividing V_max_ by [E]_t_. Correction for inner filter effects was performed as before^18^.

### LTBP4S-A cleavage assays

LTBP4S-A cleavage assays were performed as before^8^ with small modifications. Here, ADAMTS-7 –T8 (19 nM) was pre-incubated either in the absence or presence of the indicated inhibitors for 2 h at 37 °C before addition of ADAMTS7-T8 (5.6 µM). After 17 h, proteolysis was stopped by addition of Bolt^TM^ LDS Sample Buffer, 5% β-mercaptoethanol, and heating to 95 °C. Samples were frozen at 20° C before use. For SDS-PAGE analysis, 12% Bis-Tris Plus Gels (ThermoFisher) were used and stained for 6 h with Imperial protein stain (ThermoFisher). Gels were destained overnight in pure water at room temperature on an orbital shaker and scanned for analysis of LTBP4S-A cleavage bands. To accurately determine cleavage inhibition from each inhibitor, inhibition was quantified by densitometry of the bands corresponding to cleavage products using ImageJ software. The data were plotted using GraphPad Prism 7.0.

### Identification of LTBP4S-A cleavage sites

To generate LTBP4S-A cleavage fragments for mass measurement, LTBP4S-A (432 pmol) was incubated with 1.4 pmol of ADAMTS7-T8 in 25 µL TNC buffer (50 mM Tris, 150 mM NaCl, 10 mM Cacl_2_, pH 7.5) for 24 h at 37 °C. Brij-35 was left out the proteolysis reaction buffer because it interferes with the mass spectrometry (MS). Following incubation with ADAMTS7-T8, glycerol-free PNGase F (7.3 pmol, New England Biolabs) was added and incubated for 17 h. The reaction was stopped with 30 mM ethylenediaminetetraacetic acid (EDTA) and frozen at −80 °C until MS analysis. For intact mass measurement proteins were desalted using a 2 mm x 10 mm guard column (Upchurch Scientific, Oak Harbour WA) packed with Poros R2 resin (Perseptive Biosystems, Framingham). Desalted peptides were loaded on a Q-Exactive UHMR Hybrid Quadrupole-Orbitrap mass spectrometer (Thermo Fisher Scientific). The peptide solution (3 µL) was filled in gold-plated borosilicate capillaries prepared in house. Static nanospray was achieved at a resolution of 12,500 using the following: 1.2 kV capillary voltage, S-lens 100 V, m/z-range of 600–2000, HCD collisional activation of 20 V and nitrogen gas at 4 ml/min. Deconvolution of mass spectra was performed using UniDec (http://unidec.chem.ox.ac.uk/).

### *In silico* studies

The human ADAMTS-7 model was generated using the crystal structure of ADAMTS-5 bound to Batimastat as a template (PDB code 2RJQ). The protein was then subjected to 55 ns of molecular dynamic (MD) simulation as already reported^19^ and, for the last 50 ns, a protein structure snapshot was collected for each nanosecond to obtain 50 protein conformations. In order to partially take into account the side-chain flexibility of the residues belonging to the binding site, EDV33 was docked into the 50 ADAMTS7 protein conformations using GOLD software with four different fitness scoring functions included (GoldScore, ChemScore, ASP and ChemPLP). For each docking calculation, the best ranked solution was considered, thus obtaining a total of 200 docking solutions for the compound. These docking poses were then clustered and clusters with a population of at least 50 poses were considered. A root-mean-square deviation (RMSD) of 2.0 Å was chosen as a threshold. As a result, two clusters of docking poses were considered (Supplementary Figure 4).

In order to further investigate the two potential binding modes of EDV33, the two clusters were subjected to 55 ns of MD simulation. As shown in Supplementary Figure 5, the docking pose of cluster 1 is highly stable, with an average RMSD of about 1.3 Å. After about 12 ns, the docking pose of cluster 2 converged on the pose of cluster 1, supporting the reliability of the final docking disposition.

The TIMP-4 model was generated by homology modelling with HHpred and MODELLER using the structures of TIMP-1, TIMP-2 and TIMP-3 as templates (PDB codes 2E2D, 3V96, 3CKI, 3MA2)^20–22^.

## Results

### Identification of ADAMTS-7 cleavage sites in LTBP4

**Figure 1.**
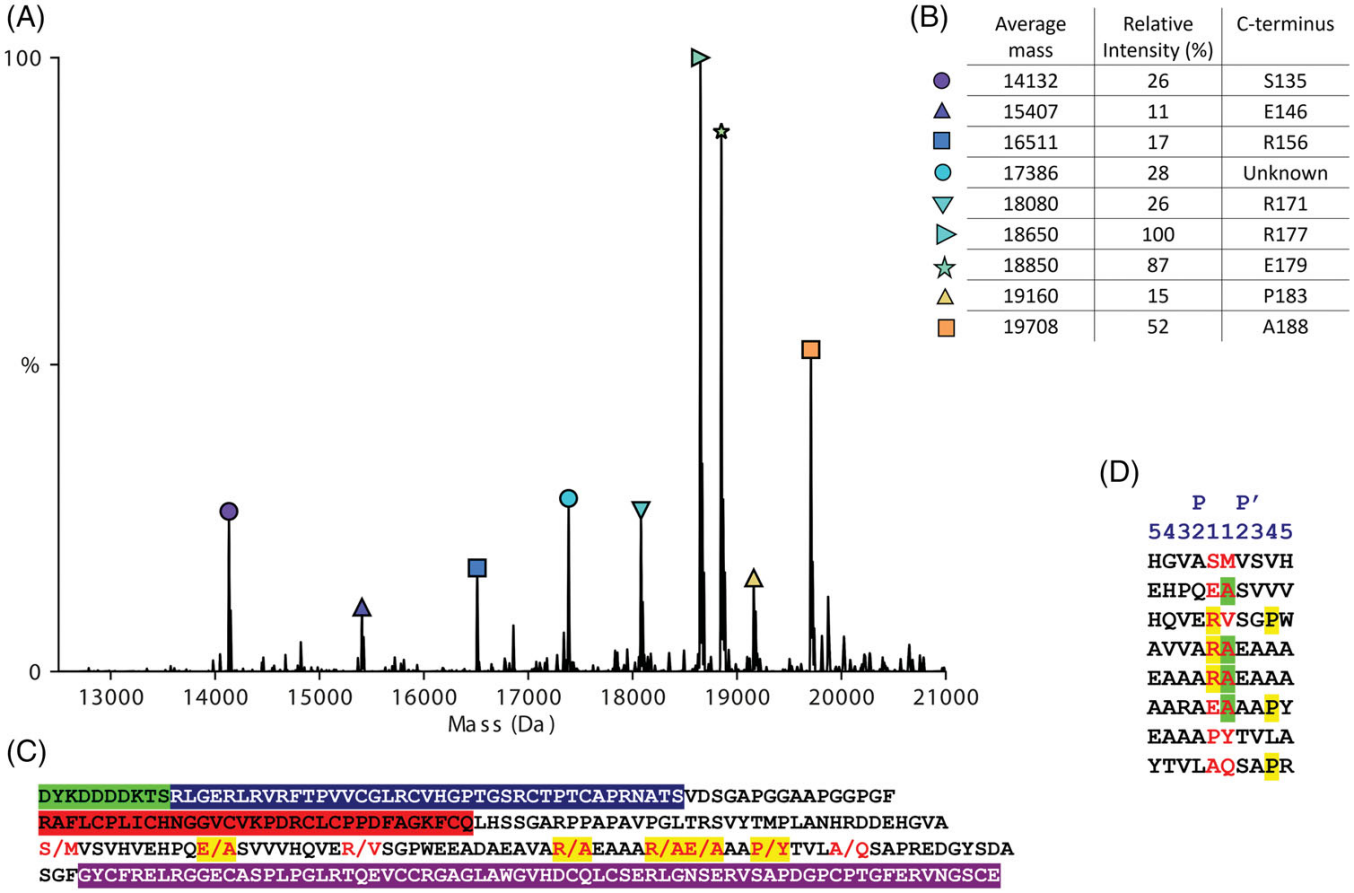
Mass spectrometry of the recombinant protein construct LTBP4S-A cleaved by ADAMTS-7. (A) Mass spectra of LTBP4S-A cleavage fragments showing the average mass in Dalton (Da) and relative peak intensities as a percentage of the highest peak. Cysteine residues were oxidised (B) Eight out of nine peaks shown could be matched to a cleavage fragment. The identified fragments all start with Asp^1^ of the N-terminal FLAG tag and have a C-terminus generated by ADAMTS-7 cleavage (P1 of the cleavage site). The relative peak intensities are an indication of the relative amounts best described as semi-quantitative. (C) Amino acid sequence of the recombinant protein construct LTBP4S-A. The residues of which the peptide bonds were cleaved by ADAMTS-7 are shown in red. Cleavage sites that we identified previously using a different method^8^ are highlighted in yellow. The N-terminal FLAG tag is highlighted in green, the 4-cys domain in blue, the EGF-like domain in red and the hybrid domain in purple. (D) Cleavage sites in LTBP4S-A established in this study. The amino acid sequences surrounding the scissile bonds identified in this study by native MS are shown. The amino acids in P1 and P1′ that form the scissile bond are shown in red. Arginine residues in P1 and proline residues in P4′ are highlighted in yellow. Alanine residues in P1’ are highlighted in green.

With the aim to identify a suitable ADAMTS-7 FRET substrate, we tested initially if FRET substrates designed for ADAMTS-4 (5,6 fluorescein [FAM]-AE↓LQGRPISIAK-carboxytetramethylrhodamine [Tamra])^18^^,^^23^ and ADAMTS-5 (FAM-TESE↓SRGAIYKK-TAMRA)^24^ could be cleaved by ADAMTS-7 at low nM enzyme concentrations, which was not the case (data not shown). As ADAMTS-7 is also prone to autolysis^8^, we had also designed a FRET substrate based on autolytic cleavages in ADAMTS-7 (TAMRA-IRIQE ∼ VAE ∼ AANK-FAM), which was also not cleaved at low nM enzyme concentrations (data not shown). This prompted us to design a new FRET substrate based on peptide bonds cleaved in LTBP4. To achieve this, we aimed to identify a short amino acid sequence that was susceptible to proteolysis by ADAMTS-7. The maximum length of a FRET substrate is dictated by the optimal distance between fluorophore and quencher on either side of the peptide substrate that allows for efficient FRET (between 15 and 60 Å or 4–17 residues)^25^. To design a short and efficient peptide substrate, information on the preferential cleavage sites of ADAMTS-7 was needed. We previously identified cleavage sites in LTBP4S-A, using tandem mass tag (TMT)-labelling of neo N-termini generated by ADAMTS-7 cleavage prior to serine protease digest and liquid chromatography (LC) tandem mass spectrometry (MS/MS) analysis^8^. To confirm these cleavage sites using a different method, we measured the exact mass of the cleavage fragments by MS. This required PNGase treatment of LTBP4S-A cleavage products to remove N-linked glycans, which are heterogeneous in mass. The MS analysis of LTBP4S-A cleaved by ADAMTS-7 showed nine major cleavage products, eight of which could be identified and matched to an ADAMTS-7 cleavage site (Figure 1). All identified cleavage products started at the original N-terminus following signal peptide removal (Asp1 of the FLAG-tag) and contained a new C-terminus generated by ADAMTS7 cleavage. Of the eight cleavage sites identified here, five of them had been identified previously [8]. The peak intensity of the protein fragments indicated that the two most abundant cleavage fragments ended at Arg^177^ and E^179^ respectively, resulting from proteolysis by ADAMTS-7 at the ^177^Arg↓Ala^178^ and ^179^Glu↓Ala^180^ bonds of LTBP4S-A respectively. These two preferred cleavage sites are numbered ^193^Arg↓Ala^194^ and ^195^Glu↓Ala^196^ in LTBP4S (Uniprot ID Q8N2S1, Isoform 2) and we will use this numbering henceforth when describing cleavage sites.

### Design of a FRET ADAMTS-7 substrate

**Figure 2.**
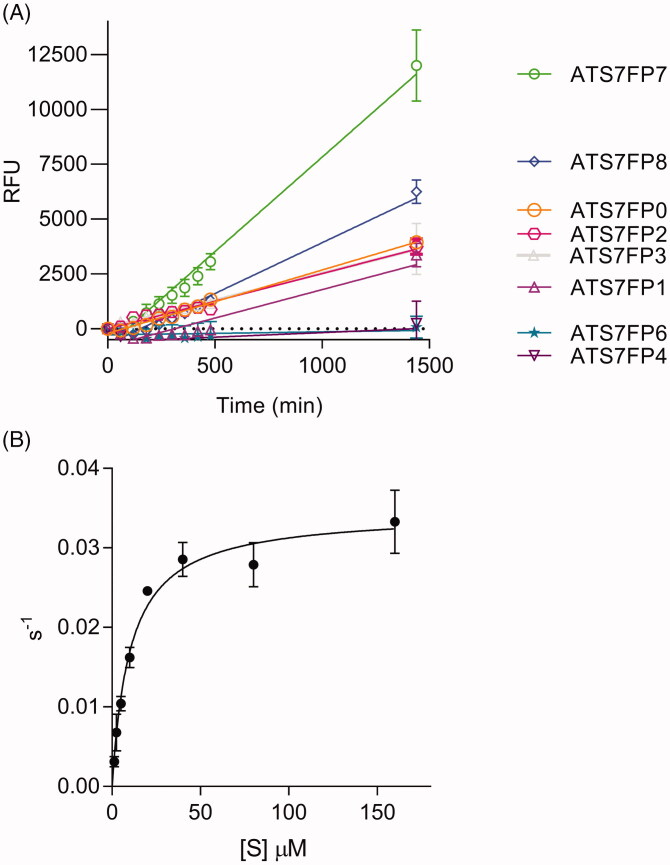
ATS7FP7 is cleaved efficiently by ADAMTS-7. (A) Cleavage of FRET peptides based on LTBP4 by ADAMTS-7. FRET peptides (40 μM) were incubated with ADAMTS-7 (10 nM). Fluorescence was detected (λ_ex_ = 485 nm, λ_em_ = 520 nm) for 24 h at 37 °C and reported as relative fluorescence units (RFU). The data are presented as average ± SEM (*n* = 3) and were fitted to a linear regression using Graphpad Prism (B) Cleavage of FRET peptide ATS7FP7 by ADAMTS-7 (10 nM) is shown. Data were fitted to the Michaelis–Menten equation and are presented as average ± SEM (*n* = 3).

The cleavage sites ^193^Arg↓Ala^194^ and ^195^Glu↓Ala^196^ were chosen to design the FRET substrate ATS7FP0 (Table 1). This peptide covers residues Ala^188^-Ala^198^ in LTBP4S and contains a FAM/TAMRA fluorophore/quencher pair. The quencher is covalently linked to the ε amino group of an extra C-terminal lysine residue. Stock solutions were routinely prepared at 10 mM in DMSO and stored at −80 °C. Upon dilution of the parental peptide, ATS7FP0, in the assay buffer, the solution appeared non-homogenous, suggesting solubility was not optimal. This was most likely due to the high content in hydrophobic amino acids of this peptide, which added to the hydrophobicity of the fluorophore/quencher pair. Nevertheless, ATS7FP0 generated fluorescence upon cleavage by ADAMTS-7 (Figure 2(A)). With the aim to improve both solubility and cleavage rate by ADAMTS-7, seven additional peptides were designed using the sequence of ATS7FP0 as a starting point. The N-terminal alanine residue present in ATS7FP0 was removed to generate peptide ATS7FP7 with a reduced aliphatic index (Table 1). Our MS analysis of LTBP4S-A had identified eight cleavage sites of which three were associated with a proline in P4′ (Figure 1(D)), suggesting a potential preference of ADAMTS-7 for proline in P4′. To assess the effect of a P4′ proline residue on the cleavage rate, peptide ATS7FP7 was modified to generate ATS7FP8. This was further modified by mutating one alanine residue into serine with the aim of improving solubility, thus generating peptide AT7FP1. Since we also determined that ADAMTS-7 prefers arginine in P1 (Figure 1(D)), we mutated one alanine residue in the parental sequence into arginine with the aim to improve both cleavage rate and hydrophilicity, thus generating peptide ATS7FP3. Mutation of both alanine residues in ATS7FP8 (into serine and arginine, respectively) generated peptide ATS7FP6. Moreover, since our results suggested a possible preference for a proline in P4′ (Figure 1(D)) we mutated the fourth alanine residue C-terminal to the Arg↓Ala cleavage site into proline (peptide ATSFP2). Finally, peptide ATSFP4, a shorter version of ATSFP2, was designed.

**Table 1.** Sequences and physical parameters of ADAMTS-7 FRET substrates.

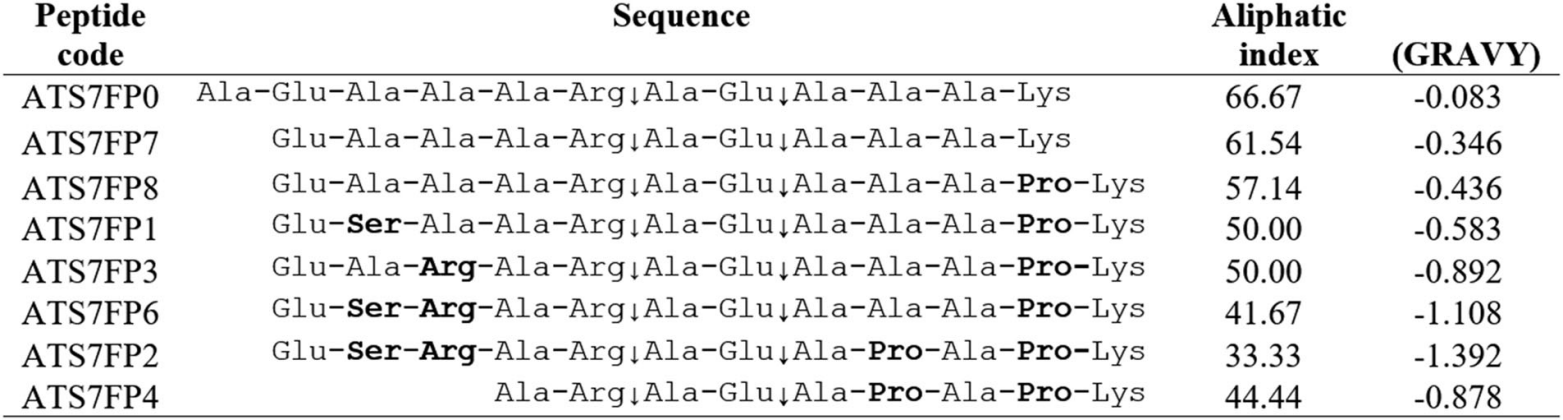			

Aliphatic index and Grand average of hydropathicity (GRAVY) were computed using ProtParam. Changes compared to the parental LTBP4 sequences are in bold, putative cleavage sites are indicated by the symbol “↓”.

Upon dilution of ATS7FP7 in the assay buffer it became apparent that removal of the alanine at the N-terminus of the parent peptide ATS7FP0 was sufficient to improve solubility, as the solution was now homogeneous, in contrast to ATS7FP0. Analysis of the cleavage rate of ATS7FP7 showed that it was increased compared to that of ATS7FP0 (Figure 2(A)), probably due to increased solubility. All other peptides were cleaved at a lower rate compared with ATS7FP7 (Figure 2(A)), likely because the introduced mutations negatively affect recognition by ADAMTS-7.

As ATS7FP7 was cleaved at the highest rate, we subsequently determined its specificity constant (*k*_cat_/*K*_m_), Michaelis-Menten constant (*K*_m_), and turnover number (*k*_cat_) for cleavage by ADAMTS-7 (Figure 2(B) and Table 2). The relatively high *k*_cat_/*K*_m_ of ∼3.4 × 10^3^ M^−1^ s^−1^ indicates that ATS7FP7 is efficiently cleaved by ADAMTS-7.

**Table 2.** Kinetic parameters for cleavage of ADAMTS-7 FRET substrate ATS7FP7 by ADAMTS-4, -5, and -7. Data are presented as average ± SEM; *n* = 3.

Enzyme	*k*_cat_ (s^−1^)	*K*_m_ (μM)	*k*_cat_/ *K*_m_ (M^−1^s^−1^) × 10^3^
ADAMTS-4	0.116 ± 0.0060	9.5 ± 1.7	13.4 ± 3.4
ADAMTS-5	0.225 ± 0.042	81 ± 24	3.0 ± 0.44
ADAMTS-7	0.035 ± 0.0017	10.5 ± 1.5	3.4 ± 0.36

We also tested if ATS7FP7 was cleaved by other ADAMTS family members. At the same concentration (10 nM), neither ADAMTS-1 nor ADAMTS-8 were able to cleave ATS7FP7. In contrast,both ADAMTS-4 and ADAMTS-5 cleaved ATS7FP7 efficiently (Supplementary Figure 6 A). Specificity constants for ADAMTS-4 and −5 were 13.4 × 10^3^ and 3.0 × 10^3^ M^−1^s^−1^, respectively (Supplementary Figure 6B, C, and Table 2). However, in the case of ADAMTS-5 a caveat is that an accurate determination of *V*_max_ was not possible as the Michaelis–Menten curve did not reach a proper plateau and substrate concentrations higher than 160 µM cannot be tested due to significant inner filter effects, as previously reported^18^.

Overall, these results show that although our novel FRET peptide ATS7FP7 is not selective for ADAMTS-7, it is cleaved efficiently by ADAMTS-7 and is an excellent tool to screen inhibitors using purified ADAMTS-7.

### Testing ADAMTS7 inhibitors

**Figure 3.**
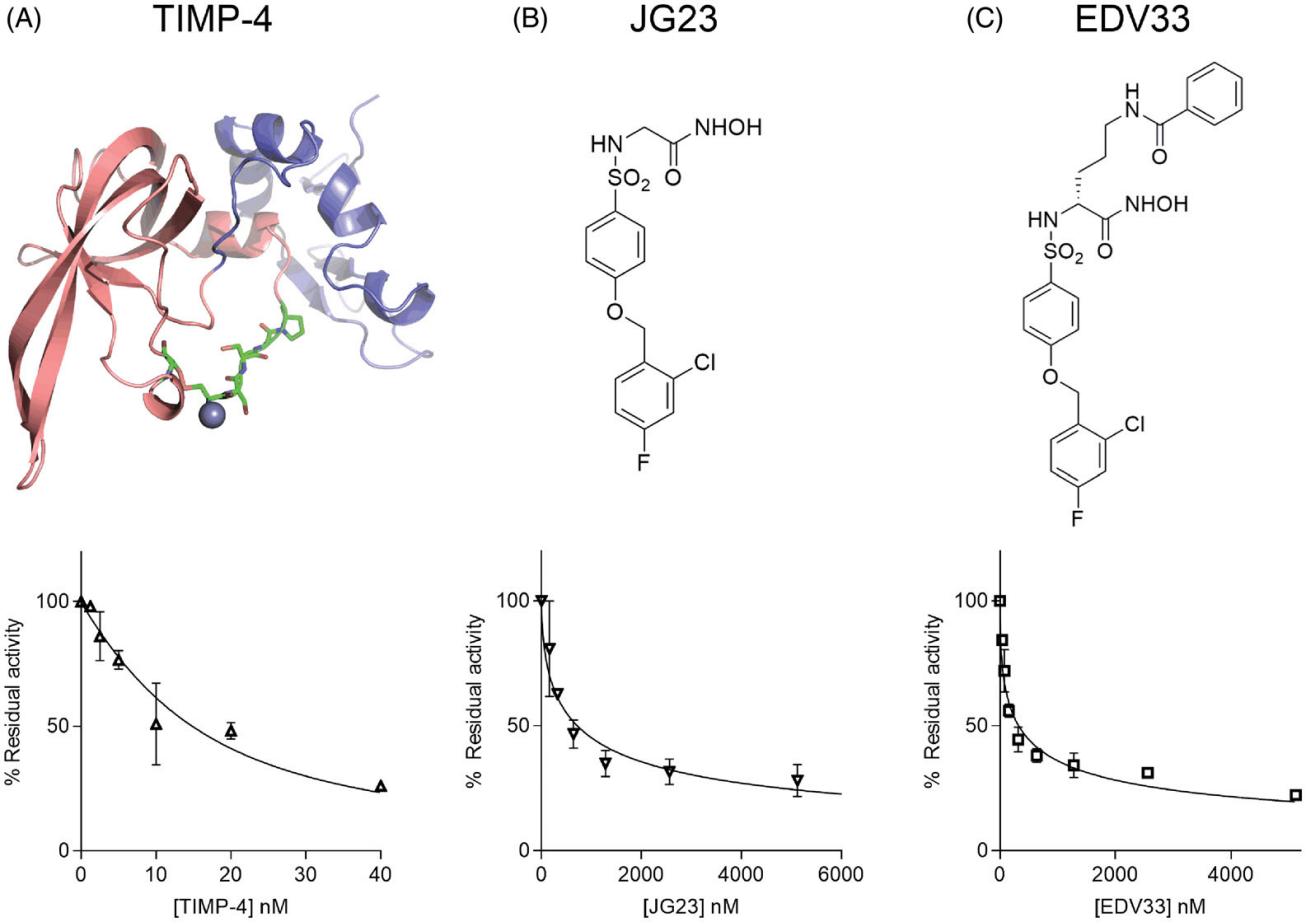
Inhibition of ADAMTS-7 cleavage of ATS7FP7 by TIMP-4, JG23, and EDV33. ADAMTS-7 was incubated for 1 h either at 5 nM with TIMP-4 (A), JG23 (B) or EDV33 (C) at 10 nM before addition of ATS7FP7 (40 μM). Percent of inhibition was calculated from control reactions containing only DMSO. For TIMP-4, data were fitted to the Morrison equation, while in case of JG23 or EDV33 the IC_50_ equation was used. The data are presented as average ± SEM (*n* = 3). Structures of TIMP-4, JG23 and EDV33 are shown above the graphs. In (A), the model of TIMP-4 was generated using homology modelling with HHpred and MODELLER. The N-terminal domain is shown in pink, the C-terminal domain in blue. Residues that sit in the active site of metalloproteases are shown as sticks with carbon in green. From left to right: Cys^102^ disulphide bonded to Cys^30^ (first residue in the mature protein), Ser^31^-Pro^34^. Chelated Zinc (Zn^2+^) in the active site of metalloproteases is shown as a blue sphere.

To the best of our knowledge, ATS7FP7 is the first reported FRET substrate for ADAMTS-7 and offers a great opportunity to test inhibitors which could potentially be employed to reduce the risk of CAD. We have recently identified TIMP-4 as an efficient endogenous inhibitor of ADAMTS-7, which inhibits cleavage of LTBP4 with a *K*_i app_ value of 13 nM^8^. We then tested the ability of TIMP-4 to inhibit ADAMTS-7 peptidolytic activity using ATS7FP7 as a substrate. TIMP-4 inhibited ADAMTS-7 with a *K*_i app_ value of 3.2 nM (Table 3 and Figure 3(A)). These data corroborate our previous finding that TIMP-4 is a potent inhibitor of ADAMTS-7 and can be used to measure concentrations of active ADAMTS-7 by active-site titration^8^.

**Table 3.** Inhibition constants and IC_50_ values for inhibition of ADAMTS-7 by hydroxamates and TIMP-4.

Inhibitor	ATS7FP7		LTBP4S-A
	*IC_50_*	*K*_i app_	*IC_50_*
TIMP-4	18 ± 6.0	3.2 ± 1.0^a^	13^b^
JG23	710 ± 80	150 ± 20	590 ± 90
EDV33	330 ± 70	68 ± 14	112 ± 14

Values are reported in nanomolar. *K*_i app_ values were determined using the Cheng-Prusoff equation unless otherwise indicated. The data are presented as average ± SEM; *n* = 3.

^a^values determined at 5 nM ADAMTS-7 using Morrison equation.

^b^values from Colige et al.^8^ using 10 nM ADAMTS-7.

**Scheme 1.**
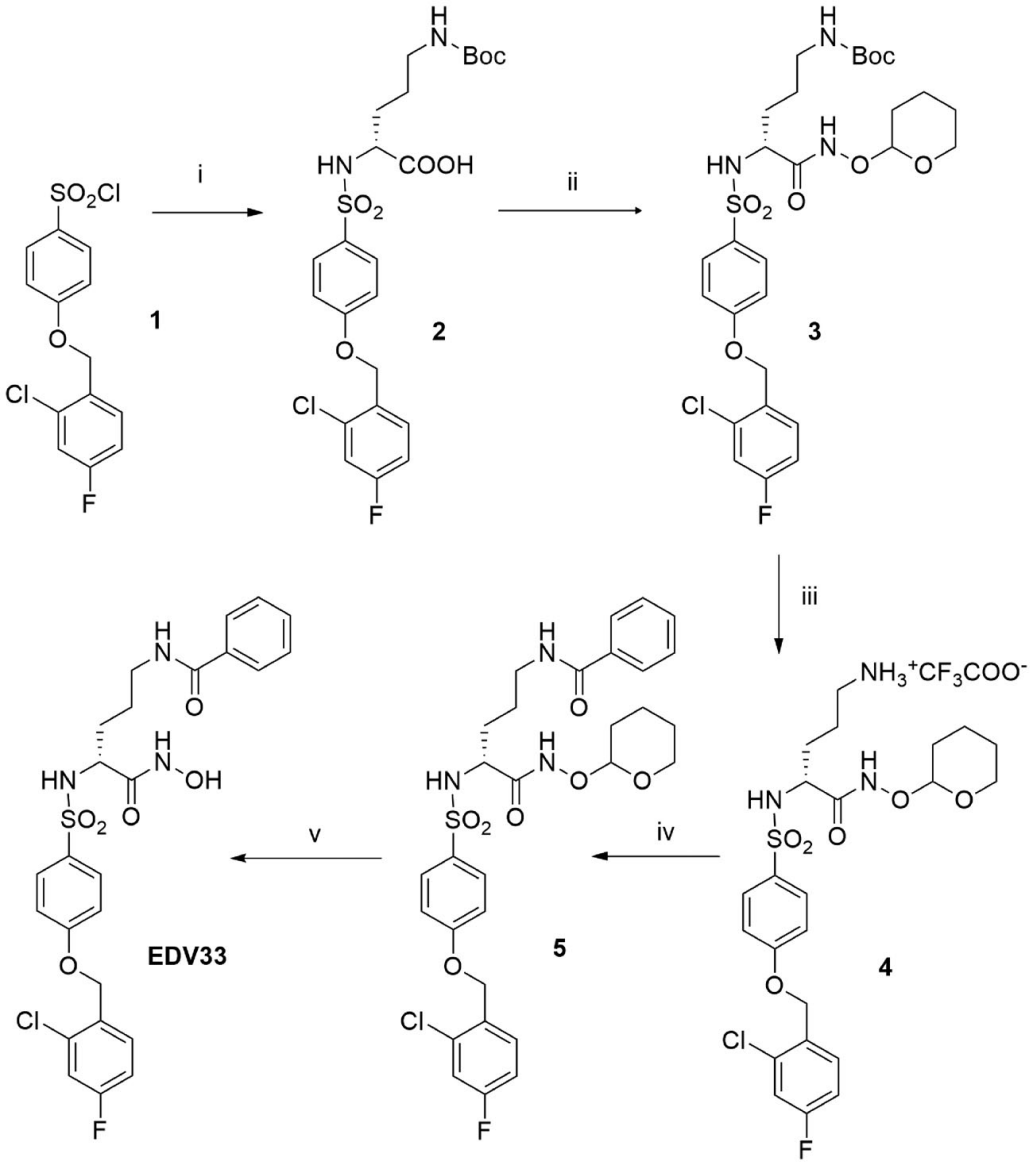
Synthesis of EDV33. Reagents and conditions: i) (*R*)-*N*-Boc-ornithine, Et_3_N, 1:1 H_2_O-Dioxane, 18 h (99.6%); ii) THPONH_2_, HOBT, NMM, EDC, DMF, 18 h (49.2%); iii) TFA, CH_2_Cl_2_, 0 °C, 30 min. (40.7%); iv) benzoyl chloride, DIPEA, DMF, 18 h (64.8%); v) 4 N HCl, dioxane, MeOH, 1.5 h (71.5%).

**Figure 4.**
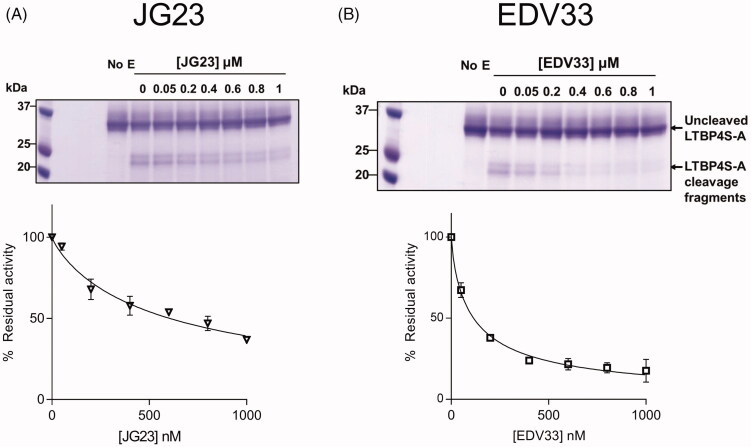
Inhibition of ADAMTS-7 cleavage of LTBP4S-A by JG23, and EDV33. ADAMTS-7 (19 nM) was incubated for 2 h with various concentrations of JG23 (A) or EDV33 (B) before addition of LTBP4S-A (5.6 μM). After 17 h, proteolysis was stopped by addition of EDTA and monitored by densitometry following SDS-PAGE/Coomassie Brilliant Blue staining. Representative gels are shown. The data in the plots are presented as average ± SEM (*n* = 3) and were fitted to nonlinear regression analysis for determination of IC_50_ values.

We then extended our analysis to small molecule inhibitors. We have previously shown that the hydroxamic acid JG23 (Figure 3(B)) inhibited ADAMTS-4 and −5 with *IC*_50_ values of 80 and 35 nM, respectively, against FRET peptide substrates^26^, and we hypothesised that it may inhibit ADAMTS-7. JG23 inhibited ADAMTS-7 activity against ATS7FP7 and LTBP4S-A with IC_50_ values of 710 and 590 nM, respectively (Table 3 and Figures 3(B) and 4(A)). The structure of JG23 was further modified by introducing an amidic chain in the α position relative to the hydroxamate (P1 group), a modification that in similar arylsulphonamidic scaffolds has been shown to increase inhibitory potency [27]. The resulting new compound was called EDV33 (Figure 3(C)). The synthesis of EDV33 has been conducted as reported in Scheme 1. The previously described sulphonyl chloride **1**^26^, was reacted with (*R*)-*N*-Boc-ornithine to give sulphonamide **2** with a good yield (>90%). (*R*)-Carboxylate **2** was firstly converted into protected hydroxamate **3** by condensation with *O*-(tetrahydro-2H-pyran-2-yl)hydroxylamine (THP-hydroxylamine) in the presence of *N*-(3-dimethylaminopropyl)-*N*'-ethylcarbodiimide hydrochloride (EDC) and then submitted to acid hydrolysis in controlled conditions to selectively remove the Boc group. Intermediate salt **4** was then acylated by treatment with benzoyl chloride to give amide **5**. Finally, the tetrahydropyran protection of **5** was removed by acid hydrolysis (4 N HCl), to afford the desired (*R*)-hydroxamic acid EDV33.

The modification of JG23 to EDV33 increased ADAMTS-7 inhibition approximately 2-fold against ATS7FP7 and 5.1-fold against LTBP4S-A (Table 3 and Figures 3(C) and 4(B)). These data demonstrate that ATS7FP7 can be used as an ADAMTS-7 substrate and that inhibition data obtained with ATS7FP7 can be extrapolated to protein substrates such as LTBP4.

**Figure 5.**
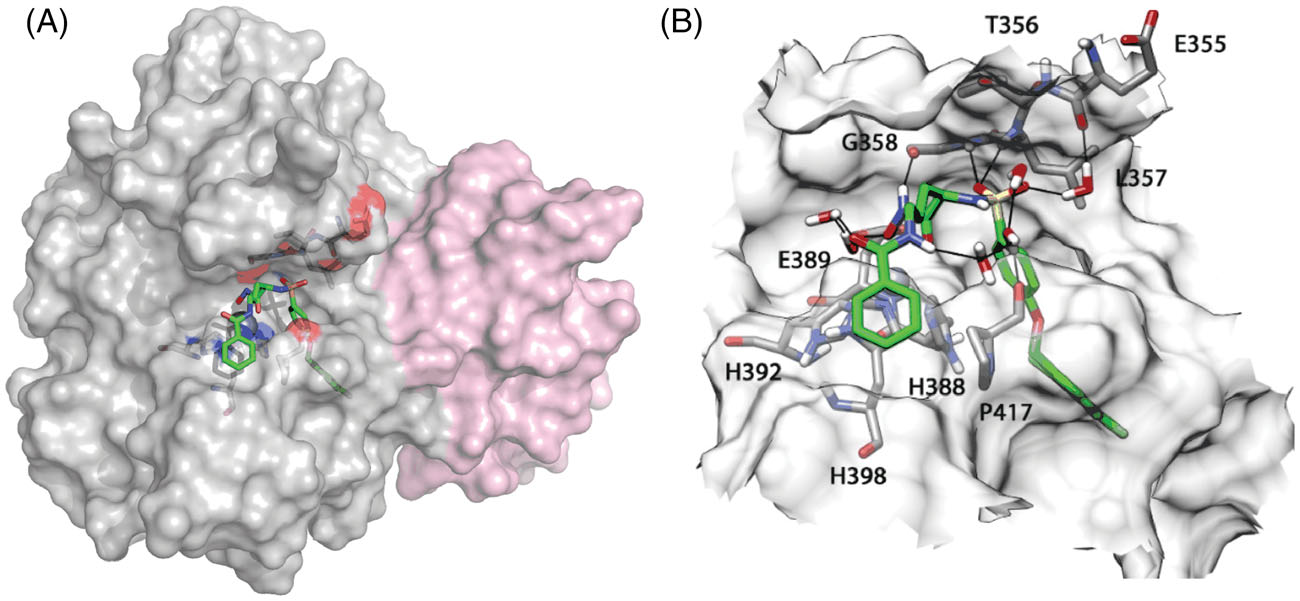
Result of the molecular dynamics simulation and proposed binding mode of EDV33 into the ADAMTS-7 metalloproteinase domain. (A) Homology model of the ADAMTS-7 metalloprotease domain (grey) and disintegrin-like domain (pink). The hydroxamate group binds to the zinc ion (Zn^2+^) in the active site (blue sphere). EDV33 is shown with green carbon atoms. (B) Zoom in of the active site. The surface of the active site is shown in a transparent representation, showing EDV33 and key interacting residues. ADAMTS-7 residues are shown with grey carbon atoms, red oxygen atoms and blue nitrogen atoms. The amino acid numbering starts from the first methionine in the signal peptide (UniProt ID Q9UKP4). For molecular dynamics simulation, see also Supplementary Figures 4 and 5.

Since we determined the *K_m_* for cleavage of ATS7FP7 by ADAMTS-7 (Table 2) and it is known that hydroxamates generally act as competitive inhibitors, it was possible to obtain *K_i_*_app_ values using the Cheng-Prusoff equation^16^ (Table 3). EDV33 inhibited ADAMTS-7 with a *K*_i app_ of 68 nM. The selectivity profile of EDV33 is reported in Table 4. EDV33 is a sub-nanomolar inhibitor of MMP-12 and a nanomolar inhibitor of the other metzincins tested. To investigate the binding mode, the metalloprotease domain of ADAMTS-7 was modelled based on the crystal structure of ADAMTS-5 (PDB code 2RJQ) (Figure 5(A)). EDV33 was docked into this new model to predict how it produces its inhibitory effect. Figure 5(B) shows the proposed binding mode of EDV33 to ADAMTS-7. The 2-chloro-4-fluoro-1-phenoxymethylbenzene fragment is inserted into the S1' cavity showing lipophilic interactions, whereas the benzamide group shows a strong lipophilic interaction with one of the three histidine residues that chelate the zinc ion (His^398^) and a lipophilic interaction with Pro^417^. Furthermore, the amidic portion is involved in water-mediated interactions with the hydroxamic acid of the ligand and the oxygen backbone of Pro^417^. Finally, the hydroxamic portion chelates the zinc ion whereas the two sulphonyl oxygen atoms establish hydrogen bonds with the nitrogen backbone of Leu^357^ and Gly^358^ and a water-mediated interaction with Glu^355^. Future compounds will need to form more extensive interactions with ADAMTS-7 pockets to improve selectivity.

**Table 4.** Selectivity profile of EDV33 against other metzincins using FRET peptides.

ADAMTS-4	ADAMTS-5	MMP-2	ADAMTS-7	MMP-12	ADAM-17
31 ± 1	6.5 ± 0.1	4.0 ± 0.3	68 ± 14	0.53 ± 0.06	4.3 ± 0.1

*K*_i_ *_app_* values are reported in nanomolar. The data are presented as average ± SEM; *n* = 3.

## Discussion

In recent years, ADAMTS-7 has emerged as a promising target for treatment of CAD^28^. However, so far no inhibitory molecules targeting ADAMTS-7 have been reported. One of the obstacles hindering inhibitor screening/development has been the lack of a suitable assay for measuring ADAMTS-7 proteolytic activity.

The purpose of the present study was to develop a substrate which can be cleaved by ADAMTS-7 and used in a convenient, rapid and fully quantitative activity assay to screen potential inhibitors. To the best of our knowledge, ATS7FP7 is the first FRET peptide substrate which can be efficiently cleaved by ADAMTS-7 and therefore easily adopted in HTS assays. This will undoubtedly accelerate the identification of anti-ADAMTS-7 inhibitory molecules such as antibodies and small molecules.

We first confirmed the identity of the LTBP-4 peptide bonds that are susceptible to proteolysis by ADAMTS-7. Of the scissile bonds that we identified previously^8^, five were confirmed here; two Glu↓Ala bonds (^162^Glu↓Ala^163^ and ^195^Glu↓Ala^196^), two Arg↓Ala bonds (^187^Arg↓Ala^188^ and ^193^Arg↓Ala^194^), and one Pro↓Tyr bond (^199^Pro↓Tyr^200^) (Isoform 2 numbering, Uniprot ID Q8N2S1). The peak intensity of the protein fragments indicated that the two most abundant cleavage fragments resulted from proteolysis by ADAMTS-7 at the ^193^Arg↓Ala^194^ and ^195^Glu↓Ala^196^ sites and these were chosen to form the basis of our FRET substrate design. Our best FRET substrate was ATS7FP7, which was cleaved by ADAMTS-7 with a relatively high specificity constant (*k*_cat_/*K*_m_) of 3.4 × 10^3^ M^−1^s^−1^, comparable in magnitude to FRET substrates currently available for other metzincins^18^.

Initially, we assessed the suitability of ATS7FP7 to characterise ADAMTS-7 inhibitors by testing TIMP-4, its likely endogenous inhibitor^8^. All TIMPs (four members in humans) act through the bidentate coordination of the active site zinc by the N-terminal α-amino group and the carbonyl group of their first residue, which is invariably a cysteine, thus effectively acting as competitive inhibitors^29^. TIMP-4 inhibited ADAMTS-7 peptidolytic activity with a very low inhibition constant, thus justifying our choice of this inhibitor to determine ADAMTS-7 concentrations by active site titrations^8^^,^^11^. This method is particularly useful to circumvent batch to batch variability in activation status or enzyme purity or when enzyme concentrations are too low to be reliably measured by optical absorbance. Therefore, an ATS7FP7-based FRET assay in combination with TIMP-4 titrations also provides a useful tool to determine active ADAMTS-7 concentrations after purification of the enzyme.

We then extended our investigation to small molecule inhibitors with a similar binding mode (i.e. the bidentate coordination of the active site zinc). We tested JG23, our previously described ADAMTS-5 inhibitor^26^ as well as its derivative EDV33 and showed that IC_50_ values determined with ATS7FP7 closely approached those determined with LTBP4S-A, thus confirming the suitability of this FRET substrate for future inhibitor screening. Small differences in the *IC*_50_ values between the two assays can be explained by the relative differences in substrate concentrations. Unfortunately, the *K*_m_ value for cleavage of LTBP4S-A by ADAMTS-7 could not be determined due to the intrinsic limitations of an SDS-PAGE-based assay, so we could not directly compare the inhibition constants measured with the two assays.

JG23 and EDV33 are the first ADAMTS-7 small molecule inhibitors reported so far. Although these are not selective for ADAMTS-7^26^ (Table 4), their structure can be used as a scaffold to build a selective ADAMTS-7 inhibitor using our model of the ADAMTS-7 metalloproteinase domain (Figure 5(A)) and we are currently investigating this possibility.

Although our new FRET substrate enables testing of inhibitors, which is a major step forward, there may also be a need for a more specific ADAMTS-7 FRET substrate to study ADAMTS-7 function in a physiological context. Such a substrate most likely needs to have sufficient length to engage exosites in other domains. For example, between 32 and 22 P residues and 4–13 P' residues are required for cleavage at the ^392^Glu↓Ala^393^ bond in aggrecan by ADAMTS-4 and ADAMTS-5^30^^,^^31^. For ADAMTS-13, a 73 amino acids-long FRET substrate was designed that iengages exosites in multiple domains, including the disintegrin-like, cysteine-rich and spacer domain^32–34^. Such a long FRET substrate requires an internal fluorophore/quenching pair that does not disrupt binding to the enzyme. At present, the structural requirements for ADAMTS-7 proteolytic activity are unknown and although we can hypothesise that ADAMTS-7 uses exosites to engage its substrates similarly to ADAMTS-4, ADAMTS-5 and ADAMTS-13^9^^,^^33^^,^^34^, this has not been demonstrated so far.

In summary, we report the first FRET-substrate suitable for testing ADAMTS-7 activity and demonstrated its suitability for assessing inhibitor potency. HTS screenings based on this peptide will pave the way for the isolation of the first selective inhibitors of ADAMTS-7 which will then be validated using *in vivo* models of CAD.

## Supplementary Material

Supplemental Material

## Data Availability

Data sharing is not applicable to the paper, because all experimental procedures and data encompassing this work are included in this manuscript.
