# Supplementary material for: Development of a fluorogenic ADAMTS-7 substrate
Source: J Enzyme Inhib Med Chem. 2021 Sep 30;36(1):2160–9. doi: 10.1080/14756366.2021.1983808 (PMC8494430; doi:10.1080/14756366.2021.1983808)
Supplement: Supplemental Material [file IENZ_A_1983808_SM3570.pdf]

Supplementary Material

Development of a fluorogenic ADAMTS-7 substrate

Salvatore Santamaria[a]*, Frederic Buemi[a], Elisa Nuti[b], Doretta Cuffaro[b], Elena De Vita[b,§], Tiziano Tuccinardi[b], Armando Rossello[b], Steven Howell[c], Shahid Mehmood[c], Ambrosius P. Snijders[c] and Rens de Groot[a,d]**

[a] *Department of Immunology and Inflammation, Imperial College London, Du Cane Road W12 0NN, London, UK;* [b] *Department of Pharmacy, University of Pisa, via Bonanno 6, 56126 Pisa, Italy.*
[c] *Proteomics Science Technology Platform, The Francis Crick Institute, 1 Midland Road, NW1 1AT, London, UK*
[d]*Institute of Cardiovascular Science, University College London, 51 Chenies Mews, WC1E 6HX, London, United Kingdom*

[§] *current address: Department of Chemistry, Imperial College London, Molecular Sciences Research Hub, Wood Lane, London W12 0BZ, UK*

Correspondence: *Salvatore Santamaria (s.santamaria@imperial.ac.uk); **Rens de Groot (R.deGroot@ucl.ac.uk)

Supplementary Table 1: Parameters used to calculate $K_{i\ app}$ values using Cheng-Prusoff equation. Substrate and K_m values are in micromolar. Abz, 2-aminobenzoic acid; Dpa, N-3-(2,4-dinitrophenyl)-L-2,3-diaminopropionyl diaminopropionic amide; Fam, 5,6-carboxyfluorescein; Mca, 7-methoxycoumarin-4-acetyl, Tamra, carboxytetramethylrhodamine.

Enzyme	Substrate	[S]	K_m	Reference
**ADAMTS-4**	Fam-Ala-Glu-Leu-Gln-Gly-Arg-Pro-Ile-Ser-Ile-Ala-Lys-Tamra	0.5	23	1
**ADAMTS-5**	Abz-Thr-Glu-Ser-Glu-Ser-Arg-Gly-Ala-Ile-Tyr-Dpa-Lys-Lys	40	76	2
**MMP-2**	Mca-Lys-Pro-Leu-Gly-Leu-Dap(Dnp)-Ala-Arg-NH_2	2	>30	3
**MMP-12**	Mca-Lys-Pro-Leu-Gly-Leu-Dap(Dnp)-Ala-Arg-NH_2	2	130	4
**ADAM-17**	Mca-Lys-Pro-Leu-Gly-Leu-Dap(Dnp)-Ala-Arg-NH_2	2	26	3

Supplementary Figure 1::**^{1}H NMR of Compound EDV33** (400 MHz, DMSO-d_6).

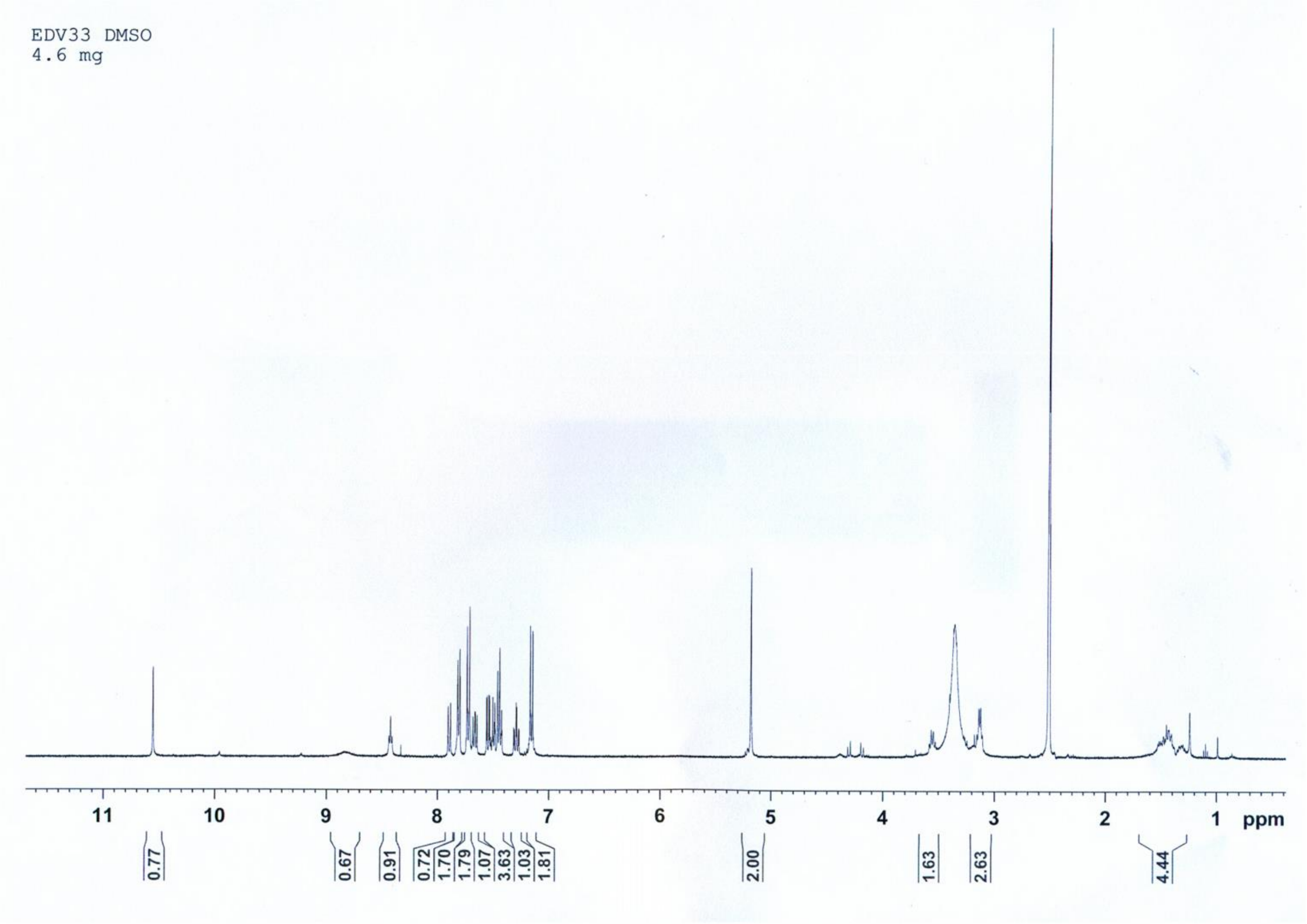

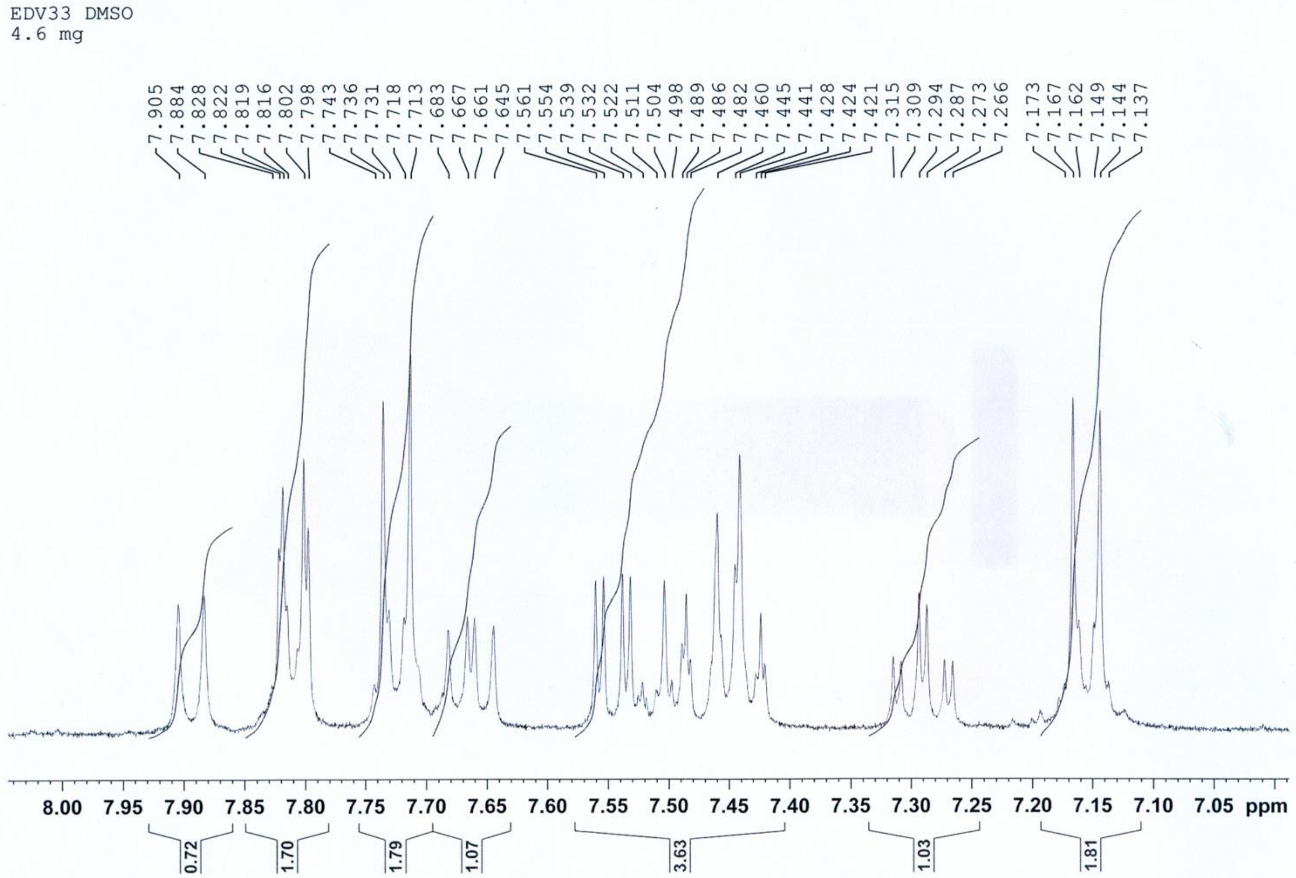
EDV33 DMSO
4.6 mg
7.905 7.884 7.828 7.822 7.819 7.816 7.802 7.798 7.743 7.736 7.731 7.718 7.713 7.683 7.667 7.661 7.645 7.561 7.554 7.539 7.532 7.522 7.511 7.504 7.498 7.489 7.486 7.482 7.460 7.445 7.441 7.428 7.424 7.421 7.315 7.309 7.294 7.287 7.273 7.266 7.173 7.167 7.162 7.149 7.144 7.137
8.00 7.95 7.90 7.85 7.80 7.75 7.70 7.65 7.60 7.55 7.50 7.45 7.40 7.35 7.30 7.25 7.20 7.15 7.10 7.05 ppm
0.72 1.70 1.79 1.07 3.63 1.03 1.81

Supplementary Figure 2: ^{13}C NMR of Compound EDV33 (100 MHz, DMSO-d_6).

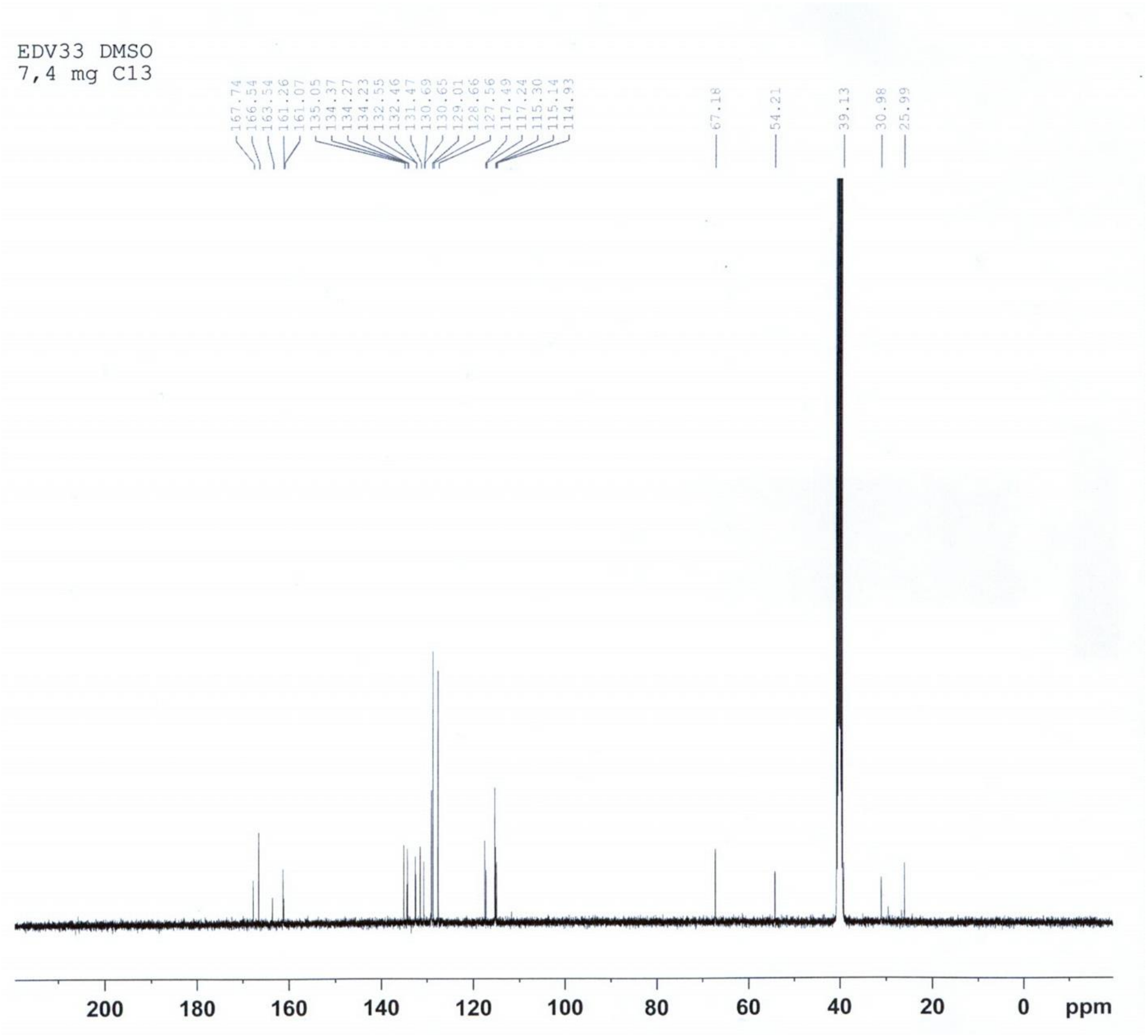

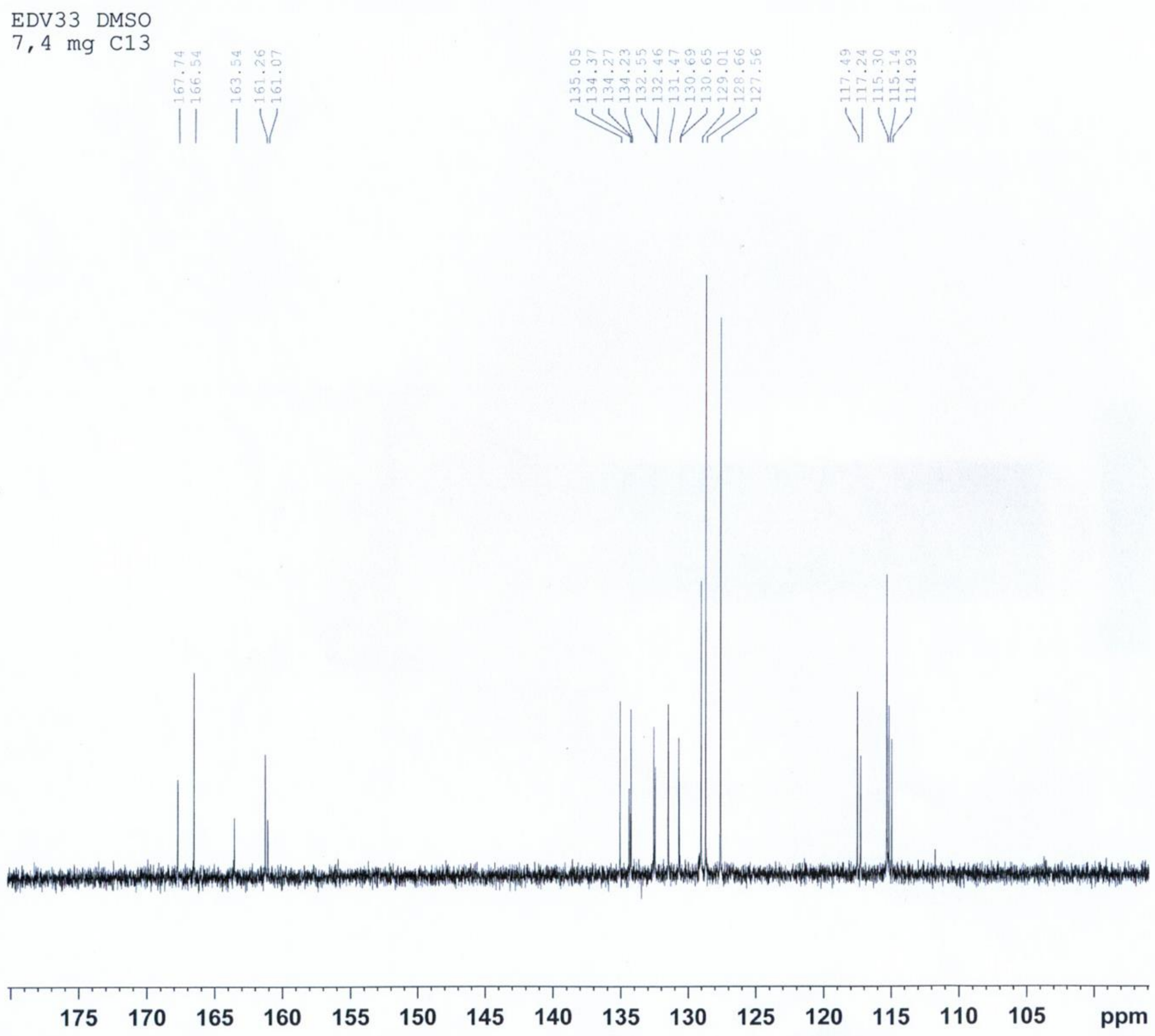

EDV33 DMSO
7,4 mg C13
167.74
166.54
163.54
161.26
161.07
135.05
134.37
134.27
134.23
132.55
132.46
131.47
130.69
130.65
129.01
128.66
127.56
117.49
117.24
115.30
115.14
114.93
175 170 165 160 155 150 145 140 135 130 125 120 115 110 105 ppm

Supplementary Figure 3: ^{19}F NMR of Compound EDV33 (376 MHz, DMSO-d_6).

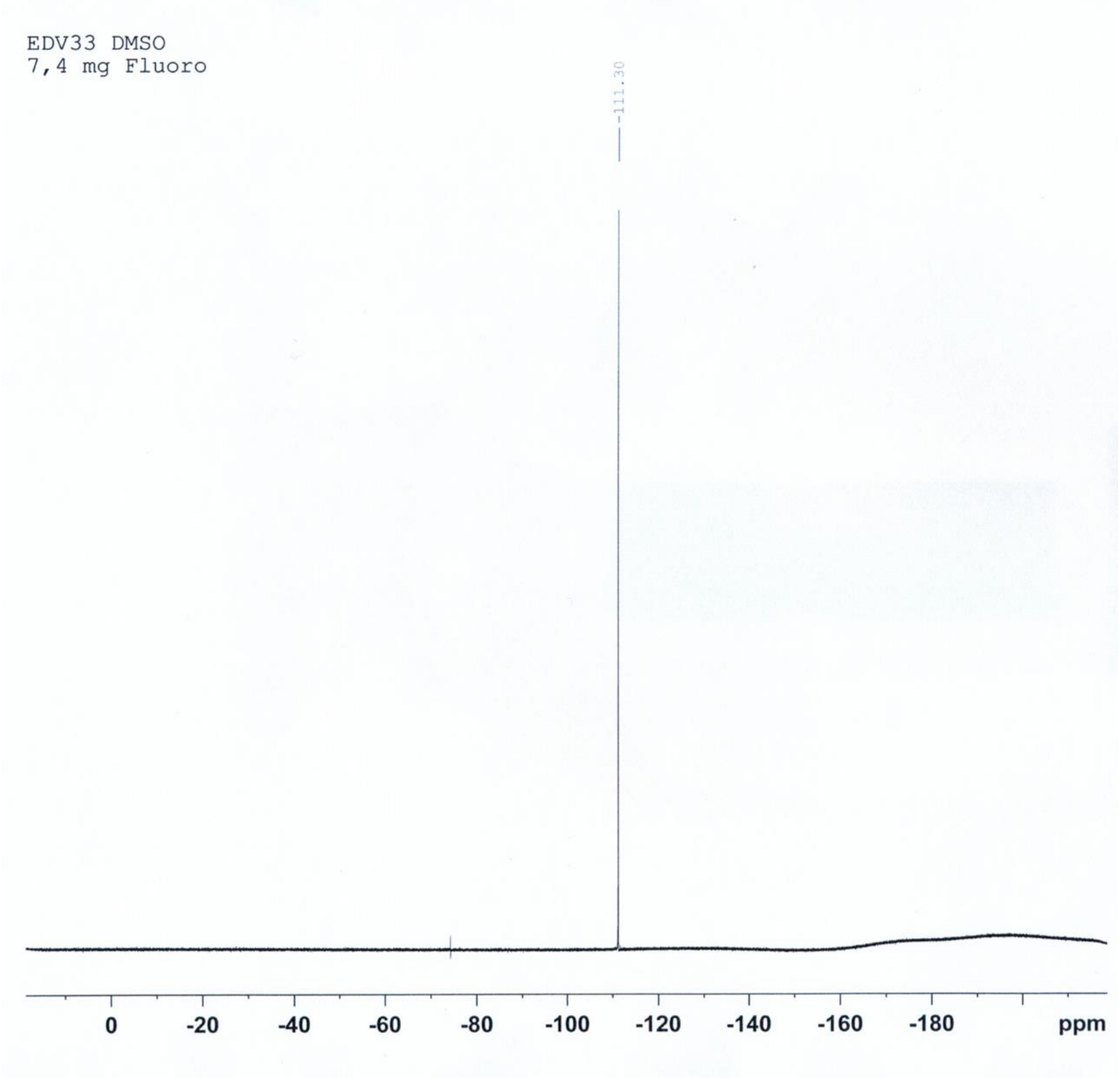

Supplementary Figure 4 – Molecular docking results for compound EDV33 bound to ADAMTS-7 (clusters 1 and 2). EDV33 was docked into 50 ADAMTS-7 protein conformations using the GOLD software with four different fitness scoring functions (GoldScore, ChemScore, ASP and ChemPLP). The best ranked docking solution from each docking calculation was considered and two clusters with a population of at least 50 poses (RMSD of 2.0Å as a threshold) were retained.

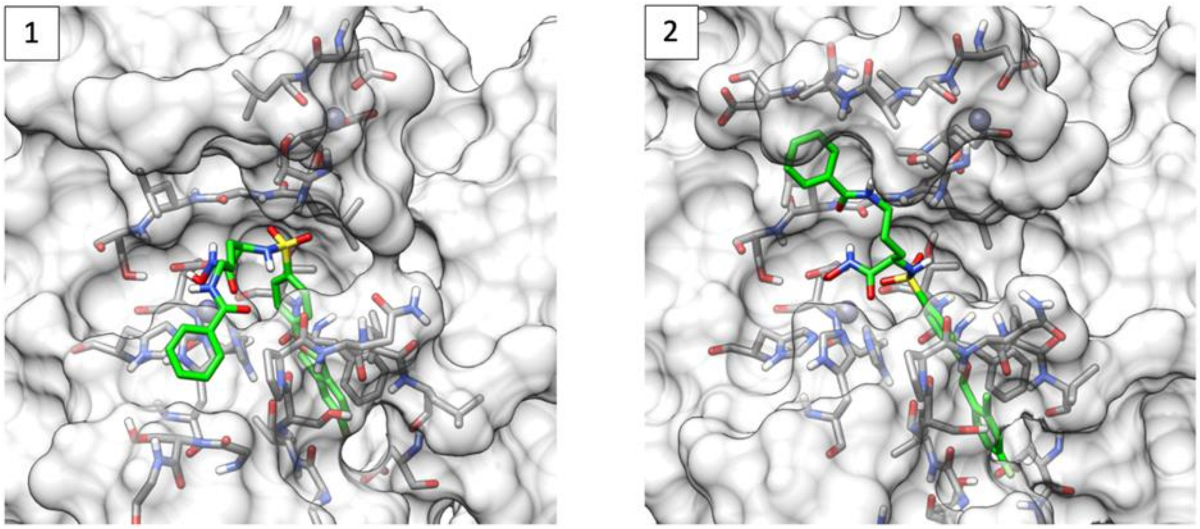

Supplementary Figure 5 – Analysis of MD simulation for compound EDV33 bound to ADAMTS-7. The plot shows the RMSD of the heavy atoms of the ligand (cluster 1 in blue and cluster 2 in orange) from the starting model structure during the simulation.

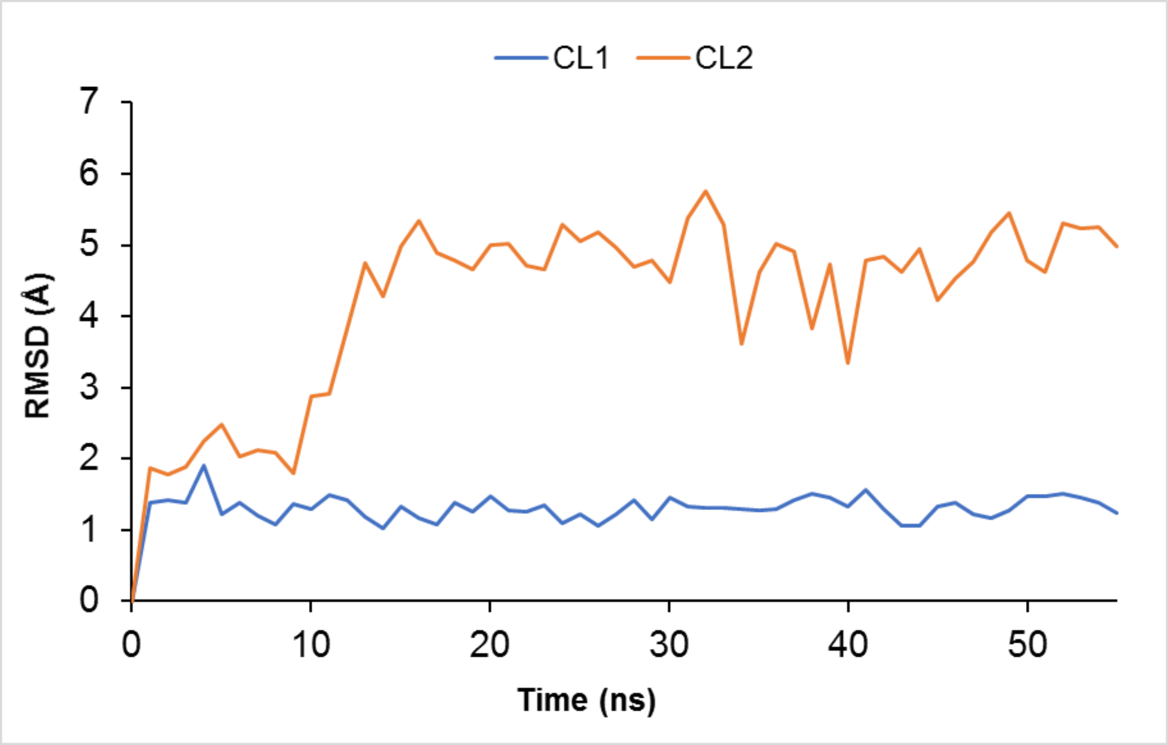

Supplementary Figure 6: Cleavage of ATS7FP7 by members of the ADAMTS family. A) ATS7FP7 (40 µM) was incubated either with ADAMTS-1, -4, -5, -7, or -8 (10 nM). Fluorescence was detected (λ_{ex} = 485 nm, λ_{em} = 520 nm) for 8 h at 37°C and reported as relative fluorescence units (RFU). The data are presented as average ± SEM (n=3) and were fitted with a linear regression. **B, C)** Cleavage of FRET peptide ATS7FP7 by ADAMTS-4 (**B**) or ADAMTS-5 (**C**) (10 nM each). Data were fitted to the Michaelis-Menten equation and are presented as average ± SEM (n=3).

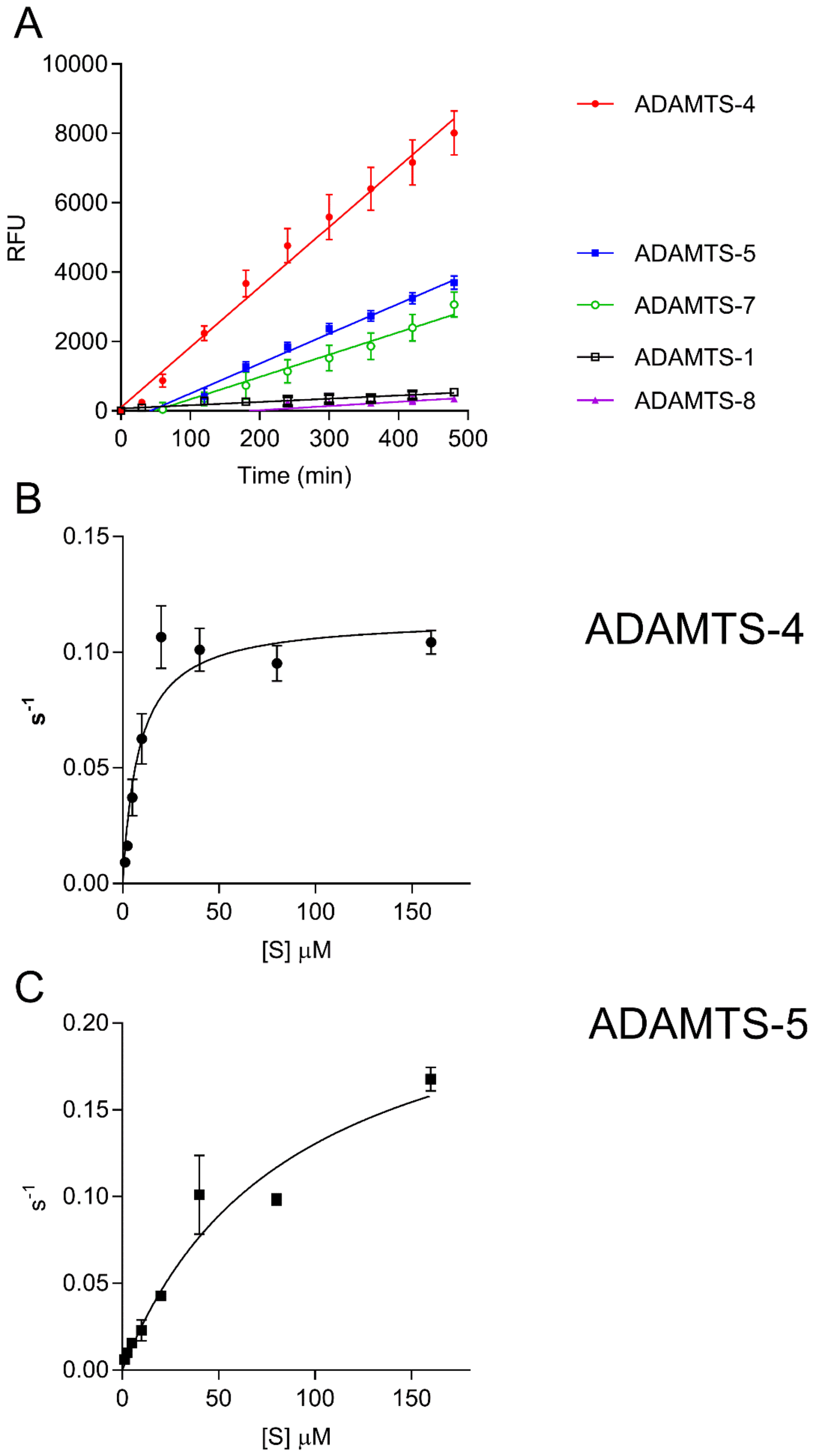
A
RFU
10000
8000
6000
4000
2000
0
0
100
200
300
400
500
Time (min)
ADAMTS-4
ADAMTS-5
ADAMTS-7
ADAMTS-1
ADAMTS-8
B
0.15
0.10
0.05
0.00
s^{-1}
0
50
100
150
[S] μM
ADAMTS-4
C
0.20
0.15
0.10
0.05
0.00
s^{-1}
0
50
100
150
[S] μM
ADAMTS-5

Supplementary References

1. Santamaria S, Nagase H. Measurement of Protease Activities Using Fluorogenic Substrates. Methods Mol Biol. 2018; 1731:107-122.
2. Troeberg L, Fushimi K, Scilabra SD, Nakamura H, Dive V, Thøgersen IB, Enghild JJ, Nagase H. The C-terminal domains of ADAMTS-4 and ADAMTS-5 promote association with N-TIMP-3. Matrix Biol. 2009 Oct;28(8):463-469.
3. Neumann U, Kubota H, Frei K, Ganu V, Leppert D. Characterization of Mca-Lys-Pro-Leu-Gly-Leu-Dpa-Ala-Arg-NH2, a fluorogenic substrate with increased specificity constants for collagenases and tumor necrosis factor converting enzyme. Anal Biochem. 2004 May 15;328(2):166-173.
4. Palmier MO, Van Doren SR. Rapid determination of enzyme kinetics from fluorescence: overcoming the inner filter effect. Anal Biochem. 2007 Dec 1; 371(1):43-51.
